# Restrictive Pattern of Pulmonary Symptoms among Photocopy and Printing Workers: A Retrospective Cohort Study 

**Published:** 2016-04-12

**Authors:** Ali Karimi, Samira Eslamizad, Maryam Mostafaee, Zahra Momeni, Fateme Ziafati, Shokoofe Mohammadi

**Affiliations:** ^a^ Department of Occupational Health, School of Public Health, Tehran University of Medical Sciences, Tehran, Iran; ^b^ Food Safety Research Center, Shahid-Beheshti University of Medical Sciences, Tehran, Iran; ^c^ Department of Occupational Health, School of Public Health, Shiraz University of Medical Sciences, Shiraz, Iran

**Keywords:** Photocopy and Printing Workers, Pulmonary Function Indexes, Respiratory Symptom

## Abstract

**Background:** According to the growth of photocopier usage in workplaces, the potential risk of occupational exposure to the airborne chemicals has been raised up. Hence, monitoring the photocopy worker's respiratory functions seems to be necessary. We aimed to evaluate the respiratory health on photocopy and printing workers so that a reliable description can be made about their occupational hygiene.

**Methods:** This study was performed in Shiraz, southwest Iran in 2014 and a group of 150 photocopy and printing workers were surveyed as exposed group in addition to a group of 114 office staff as unexposed group. The respiratory standard questionnaire was used to evaluate the prevalence of respiratory symptoms among the selected staff. Pulmonary function indexes including VC, FVC, FEV1 and the FEV1/FVC ratio were calculated. Finally, t-test, Chi Square and multiple logistic regressions were conducted.

**Results:** VC, FVC and FEV1 in photocopy and printing workers were lower than the unexposed group of which these differences for FVC and FEV1 were statistically significant (*P*<0.05). Moreover, the prevalence of all respiratory symptoms, except the shortness of breath, in exposed group was more than the unexposed group and the prevalence of coughing and wheezing was statistically significant (*P*<0.05). There was a significant difference in respiratory symptoms (cough and wheezing) between two groups after controlling for confounding variables, OR: 2.61 (95% CI: 1.21, 5.62) and 2.92 (95% CI: 1.25, 6.84), respectively.

**Conclusions:** The prevalence of excess respiratory symptoms along with pattern of pulmonary restrictive sings in photocopy and printing workers revealed that the workplace conditions can result in occupational respiratory diseases.

## Introduction

In recent decades, public concern about the adverse effects of indoor air quality on worker’s health has been noted. This growing concern is due to spending more than 90% of worker’s daily time in workplaces^1^. Furthermore, in most of the cases, concentrations of the indoor air pollutants are much higher than the outdoor ones^2^. As each worker spends one third of his lifetime at the workplaces, conditions at the workplaces can directly affect the worker's health^3^. Moreover, new electronic devices caused bad effect on health at indoor workplaces^4^.

Photocopiers, as inevitable and useful machines in offices, emit toner particulars, toxic gases including O_3_, NO_2_, non-ionizing radiation, particular matters, paper particles, nanoparticles and volatile organic compounds (VOCs)^5, 6^.

Photocopy staffs are potentially exposed to high concentrations of the photocopier emissions^7^. Katia Duarte et al. has introduced the photocopy rooms as VOC storage^8^. In Tehran, the least ratio of BTEX (Benzene, Toluene, Ethyl benzene and Xylene) in indoor/outdoor ratio (I/O) was about 42^1^. These conditions can be due to the use of photocopiers as source of emission pollution. More than 60 kinds of different VOCs are emitted to the air during using the photocopiers^1^. VOCs can be emitted while toner combining and mixing and while heating the papers. Polymers and printer's electronic components can be named as other sources of pollution as well^9^.

VOC exposure can lead to acute and chronic effects on respiratory health, neurotoxicity, lung cancer, eye and throat irritations^2^. Hence, photocopy and printing centers are so important to be monitored because the workers spend hours working around the photocopiers^10^. Moreover, health complaints (especially about the respiratory system, nervous system and immune system) associated with occupational exposure to the photocopiers has been reported^11^. Several studies have resulted in the existence of a relationship between the chronic exposure to photocopier emissions and symptoms such as dyspnea, non-allergic rhinitis, sore throat, cough, asthma, allergic inflammation in respiratory tract, upper respiratory tract infections, skin and eye irritation, headache and sick building syndrome, etc^2, 5,10, 11^.

Related studies have reported a positive correlation between the use of a photocopiers and respiratory symptoms rate ^2, 11^. A study in Denmark has shown a relationship between airway inflammation and eye irritation in photocopier users^12^. Other similar studies have shown a positive correlation between working with photocopiers and airway irritations ^2, 10, 12^‏.

A study have shown that the main differences between the photocopy and printing centers in Iran and the developed countries are in their locations and air conditions^1^. As in Iran, most of the photocopy and printing centers are located in basements with poor air conditioning and small dimensions. Thus, this nonstandard exposure to the emissions can have adverse effects on worker’s health^1^.

The present study aimed to evaluate the respiratory health on photocopy and printing workers so that a reliable description can be made about their occupational hygiene.

## Methods

This retrospective cohort study was conducted after the determination of exposed group including all 150 photocopies and printing workers in Shiraz, southwest Iran and unexposed group including 114 male office workers in 2014. The exposed group included male workers exposed to the photocopier’s respiratory pollutants.

There were several criteria for choosing the exposed group including having one year of full-time working with photocopier, having no surgery on respiratory system and no exposure to airborne pollutants in their previous careers. A number of 114 office workers were chosen as unexposed group who had the same background with the exposed group.

The workplace conditions assessment checklist contained information such as workplace dimensions, number of workers, air conditioning, heating and cooling systems provided after observation and interviews. Three questionnaires were designed to collect information about the demographic variables, occupational hygiene and the prevalence of respiratory disorders and the spirometry function tests simultaneously. Spirometry tests included VC, FVC, FEV1 and the FEV1/FVC ratio. Tests were done on the basis of American Thoracic Society (ATS) using Fukuda Sangyo Spirometer model ST-150 made in Japan. Daily calibration was done after each 4 h in accordance with its manufacture’s guideline. Each person was taught the correct spirometry tests.

Finally, to determine the comparison between the quantities variables in two groups, after group matching (matched variables: age, height, BMI and weight) independent *t*-test was used and multiple logistic regressions to unexposed confounding variables and calculate odds ratio. Data was analyzed using SPSS 19 (Chicago, IL, USA).

## Results

Demographic data and information related to the number of smokers in photography centers (exposed group) and unexposed group is shown in Table 1.

**Table 1:** Demographic information of photocopy workers and the unexposed group

**Demographic characteristics**	**Unexposed group (n=114)**		**Exposed group (n=150)**		***P*** ** value**
**Continuous variables**	**Mean**	**SD**	**Mean**	**SD**	
Age (yr)	34.32	05.71	33.42	08.40	0.951
BMI	69.61	12.72	69.35	14.30	0.836
Work experience (yr)	08.93	05.64	12.01	07.47	0.001
**Categorical variables**	**Number**	**Percent**	**Number**	**Percent**	***P*** ** value**
Smoking status					0.001
Smoker	006	04.4	035	23.3	
Nonsmoker	108	95.6	115	76.7	

Table 2 compares the results of pulmonary function tests between two groups. As seen, pulmonary function indexes including VC, FVC, FEV1 in the exposed group was less than the unexposed group, but the differences for FVC and FEV1 indexes were statistically significant (*P*<0.05). Besides, FEV1/FVC mean in the unexposed group and the exposed group was almost the identical.

**Table 2:** Comparing respiratory functions of photocopy workers and the unexposed group

**Demographic characteristics**	**Unexposed group (n=114)**		**Exposed group (n=150)**		***P*** ** value**
	**Mean**	**SD**	**Mean**	**SD**	
Vital Capacity(VC)%	85.64	9.21	83.21	11.51	0.065
Forced Vital Capacity(FVC) %	90.52	9.30	86.93	12.53	0.008
Forced expiratory volume in 1 second (FEV1) %	91.13	9.62	87.04	12.80	0.003
FEV1/FVC%	84.78	4.63	84.97	07.24	0.790

Comparison between the respiratory symptoms in both groups can be found in Table 3. The prevalence of all symptoms except asthma, were more common among photocopy and printing workers rather than the unexposed group, although the result of Chi-square test showed that only the prevalence of cough and wheezing in photocopy and printing workers was significantly more than the unexposed group (*P*<0.05) and other prevalence of symptoms between two groups was not significantly different.

Multiple logistic regression analysis was used as control for potential confounding variables including age, work experience, smoking, BMI (Body Mass Index) and marital status (Table 3). The prevalence of coughing and wheezing in photocopy and printing workers was still significantly more than that of the unexposed group. Cough and wheeze odd ratios after controlling for potential confounding variables in the exposed group were 2.61 (95% CI: 1.21, 5.62) and 2.92 (95% CI: 1.25, 6.84) times more than in the unexposed group, respectively.

**Table 3:** Adjusted and unadjusted OR (odds ratio) of respiratory symptoms of photocopy workers in comparison with unexposed group, on the basis of multiple logistic

**Respiratory signs/symptoms**	**Unexposed** **(n=114)**	**Exposed** **(n=150)**	**Unadjusted OR** **(95% CI)**	***P*** ** value**	**Adjusted OR** **(95% CI)** ^a^	***P*** ** value**
Cough						
Absent	100	112	1.00		1.00	
Present	14	38	2.33 (1.19, 2.55)	0.012	2.61 (1.21, 5.62)	0.016
Sputum						
Absent	103	120	1.00		1.00	
Present	11	30	1.52 (0.72, 3.23)	0.181	1.31 (0.93, 1.03)	0.74**1**
Phlegmatic cough						
Absent	101	119	1.00		1.00	
Present	13	31	1.92 (0.95, 3.84)	0.069	2.05 (0.92, 4.63)	0.079
Wheezing						
Absent	111	133	1.00		1.00	
Present	3	17	4.18 (1.19, 14.6)	0.016	2.92 (1.25, 6.84)	0.013
Shortness of breath						
Absent	93	120	1.00		1.00	
Present	21	30	0.73 (0.38, 1.37)	0.321	1.32 (0.74, 2.36)	0.342
Press on chest						
Absent	97	120	1.00		1.00	
Present	17	30	1.40 (0.73, 2.68)	0.204	1.27 (0.60, 2.67)	0.520

^a^ adjusted for age, work experience, smoking, BMI and marital status

Only 5% of photocopy and printing workers were using respiratory protection apparatus, 19% of them had experienced skin problems, 15% of them had passed occupational health and safety training courses and 11% of them had regular health check-ups.

## Discussion

We aimed to evaluate the level of respiratory health among photocopy and printing workers. The percentage of workers whose FVC and FEV1 was less than normal (<80%) in exposed group was more than that of the unexposed group. In addition, spirometric results show a significant reduction in FVC and FEV1 in exposed group as compared to the unexposed group (Table 2).

Lung capacity reduction indicated a pattern of pulmonary lesions in exposed group. Due to the same reduction in FVC and FEV1, the FEV1/FVC ratio did not show a significant difference and consequently there were no differences between the exposed and unexposed groups in this issue. These findings confirmed to another study^5^ which had described the more prevalent rate of respiratory symptoms among photocopy and printing workers, although there was no significant difference between the prevalence of respiratory symptoms in their study (28% in unexposed group and 30% in exposed group, respectively). Yang CY and Huang YC have conducted a relevant study with results not showing a considerable relationship with the photocopier emissions and increase in chronic respiratory symptoms^7^.

In this study, the prevalence of respiratory symptoms such as cough, sputum, phlegmatic cough, wheezing and press on chest in photocopy and printing workers were more than the unexposed group. It is important to consider the fact that among the mentioned symptoms between two groups, only the prevalence of cough and wheezing were significantly different (Table 3) and most of the worker’s complaints were from cough. In a study carried out by Alessandro et al. on a female worker, it was proved that she had chest, pain and cough problems^13^.

However, in another study, the prevalence of sputum cases among workers in comparison with the unexposed group had been recorded ^5^. In this study, despite the other respiratory symptoms, the shortness of breath was more frequent.

Logistic regression analysis was done in order to control confounding variables (age, work experience, smoking, BMI and marital status) which demonstrated in a significant difference between wheezing and cough (Table 3). The cough and wheezing odds ratios in the exposed group were more than the unexposed group. For as much as these results are conducted after the control for confounders and with regards to other studies which confirmed high concentrations of VOC in photocopy and printing centers^14^ and the VOC sources in these centers,,^15^, we can pose a hypothesis that the main reasons for cough and wheeze and lung capacity reduction (such as FEV1 and FVC) among the photocopy and printing workers is their exposure to the photocopier's emitted pollutants. Besides, lack of adequate occupational hygiene and protections in photocopy and printing centers can culminate in respiratory problems.

The main limitation of this study was that the study population was male specific, and the potential of is that all related workers in a big city were surveyed and compared with unexposed office workers as control groups.

## Conclusions

The study has shown a restrictive lung symptoms pattern in photocopy and printing centers. Due to the considerable frequency of photocopy and printing centers and the high concentrations of VOC in those places, providing a regular control on these workplaces to protect the workers’ health seems necessary. In this regard, suitable air conditioning implementation to prevent the accumulation of pollutants in photocopy and printing centers, organizing workshops and educational courses to enhance the worker's occupational hygiene awareness, regular career check-up to monitor the worker's pulmonary function and early diagnosis of probable diseases, appropriate personal protective equipment, increasing the dimensions of photocopy and printing centers and using the natural air conditioning in case there is no mechanical air condition system, are recommended.

## Acknowledgments

The authors would like to acknowledge all who helped us to enrich the paper by filling the questionnaires.

## Conflict of interest statement

The authors declare that there is no conflict of interest regarding the publication of this paper.
